# Relationship between reduced albumin and inflammation in the critically ill

**DOI:** 10.1186/s13054-015-0746-7

**Published:** 2015-04-17

**Authors:** Marco Marano

**Affiliations:** Hemodialysis Unit, Maria Rosaria Clinic, via Colle San Bartolomeo, 50, Pompeii, 80045 Italy

## ᅟ

I read the interesting article by Zampieri and colleagues [1] reporting the new association between inflammatory cytokines and acid-base components in 87 ICU patients. I would like to note that whatever approach is used to investigate the acid-base status (the celebrated bicarbonate-centered approach, the base excess methodology or the physicochemical approach) most of the acid-base parameters have values within or slightly beyond the normal range [2,3]. Specifically, this is true for the pH, the pCO_2_ and the HCO_3_ values as well as for the standard base excess (SBE) value. As regards the physicochemical approach used by the authors, the independent variables determining the pH value show only a marginal distance, if any, from their reference value [2]. Notably, the SIDa (apparent strong ion difference) value is perfectly normal. The SIG (strong ion gap) value is above the upper normal threshold, but it should be remarked that such excess may be due, to a large extent, to the low amount of albumin (because the albumin value is one of the relevant parameters used to compute the effective strong ion difference and hence the SIG value). Therefore, the only acid-base parameter that the authors actually found to be abnormal is the albumin one. Since albumin is accepted as a basic element only in the physicochemical approach, and this approach is not unanimously adopted, the title of the article might be misleading. Concluding, it is my opinion that the innovative findings by Zampieri and colleagues should be more properly depicted as a relationship between reduced albumin and inflammation. My argument does not affect the validity of their findings and I agree with the authors that they could open a new field of research.

## Abbreviations

SBE: Standard base excess
SIDa: apparent strong ion difference
SIG: Strong ion gap
