# Supplementary material for: Relationship between reduced albumin and inflammation in the critically ill
Source: Crit Care. 2015 Apr 17;19(1):177. doi: 10.1186/s13054-015-0746-7 (PMC4403944; doi:10.1186/s13054-015-0746-7)
Supplement: Related texts

# Reply: Author’s response

**Authors:** Fernando G Zampieri, John A Kellum

We would like to thank Dr Marano for his interest in our manuscript. While we agree that the main finding in our manuscript was the potential modulation of the inflammatory response by albumin, we do not believe that our other conclusions regarding the remaining components of the acid-base status should be diminished. We think so for two reasons.

First, the normal mean values of many acid-base components found in our study do not necessarily imply that all patients had close to normal values for SIG, SIDa or SBE. In fact, they were pretty abnormal. Regarding SIG, 29 patients had SIG values higher than 10 mEq/L, which cannot be accounted for only due to low albumin [4]. The range of SIDa was 24.1 to 52.1 mEq/L (Figure 1), with a quarter of the included patients presenting with values below 37 mEq/L.

**Figure 1:** **Density plot for SIDa (apparent strong ion difference) values.**

Second, as shown in Additional file 1 of our paper, relationships were complex in nature and nonlinear. In fact, as shown in multi-adaptive analysis plots, albumin reduction *per se* was not always associated with inflammation (for example, no association was seen between low albumin and high MCP1 when SIG was low). In principal component analysis, albumin was part of both principal component 1 and 2, suggesting that it modulated the association but not necessarily was an independent factor. Thus, while we agree that albumin is important, and that our results support further research involving albumin, other acid-base variables were also important contributors to the overall findings of our study.
